# Educating Medical Students for Practice in a Changing Landscape: An Analysis of Public Health Topics within Current Indonesian Medical Programs

**DOI:** 10.3390/ijerph182111236

**Published:** 2021-10-26

**Authors:** Nurhira Abdul Kadir, Heike Schütze, Kathryn M. Weston

**Affiliations:** 1 Faculty of Science Medicine and Health, School of Medicine, University of Wollongong, Wollongong, NSW 2522, Australia; h.schutze@unsw.edu.au (H.S.); kathw@uow.edu.au (K.M.W.); 2 Faculty of Medicine and Health Sciences, School of Medicine, Universitas Islam Negeri Alauddin Makassar, Makassar 90221, Sulawesi Selatan, Indonesia; 3 Faculty of Medicine, School of Public Health and Community Medicine, University of New South Wales, Kensington, NSW 2033, Australia

**Keywords:** public health, medical education, medical curricula

## Abstract

Medical curricula need to prepare doctors for emerging health issues and increased public health roles. With medical schools spread over a vast geographical region of Indonesia, ensuring that all schools meet appropriate standards in the quality of subjects, course delivery, and performance is challenging. This paper explores the inclusion of public health subjects in medical education across the country. A search of all subjects (n = 388) who were taught in 28 representative medical schools was undertaken and categorized by geographical region, accreditation grade, and according to the Indonesian National Standard of Medical Competency. Basic biomedicine subjects had the highest representation in the curricula (49.2 ± 8.7%) and public health was generally well represented (14.3 ± 5.0%). All medical schools complied with the minimum of 144 credits required for the bachelor stage. No statistically significant difference was found between school accreditation grades, or when an overall comparison of programs in Eastern and Western regions was undertaken. The Indonesian medical schools included have relatively good curriculum transparency, and public health is an important feature in their curricula. Further research is critical to identify the materials taught, the relevance and the applicability of the specific public health content, and the assessment of public health competency of graduates.

## 1. Introduction

Medical doctors are integral to healthcare systems in many ways; they contribute not only as clinicians and evidence-based practitioners, but also as leaders, advocates, and health system reformists, working to improve health care provision and equity [1,2]. Within the broad sphere of public and population health, medical doctors play a key role at the local and community level in disease prevention and health promotion, as well as being influential at a national or administration level through direct involvement in the development of policy [3,4]. Their roles and responsibilities are increased during times of national and global public health threats, such as the current COVID-19 pandemic, as doctors are expected to be flexible and adaptable and to take on increased workloads and responsibilities, including public health aspects of disease prevention and mitigation, outbreak management, and other public health roles [5].

In the context of medical education, public health in this paper refers to aspects of population health including health promotion, environmental health, and prevention of disease, as opposed to government-funded tertiary health services (often called public health services). Medicine continues to be dominated by a biomedical worldview whereby diseases are treated in isolation from social determinants of health [6]. Indeed, in the global medical education sphere, it is well recognized that biomedical and clinical subjects can often overcrowd the medical curricula, leaving little or insufficient space for public health subjects [1]. This deficiency has been the subject of scholarly discussion, and many institutions worldwide have developed strategies to address or remedy this situation [6].

### The Indonesian Context

The Republic of Indonesia consists of over 17,000 islands covering almost 2 million square miles in Southeast Asia and Oceania. With a population over 270 million, Indonesia’s diverse environment and society includes forested volcanic mountain regions, coastlines, and rural and urban communities that include over 1300 ethnic groups and over 700 languages. Although emerging as a global market economy, Indonesia is currently categorized as a low- and middle-income country (LMIC) and has complex public health issues [7]. Among its health priority areas are the following: high maternal and child death rates, which are among the highest in Southeast Asia (305 maternal deaths per 100,000 live births and 24 child deaths per 1000 live births); high mortality and morbidity due to infectious diseases (for example, the death rate for HIV-negative tuberculosis in 2019 was 34 deaths per 100,000 population, which was the highest rate in Asia [8]); childhood stunting (with rates as high as 41.2% in some provinces); and high rates of non-communicable diseases [7,9,10].

In order to ensure continued improvement in health care and service provision across Indonesia’s priority health areas, and thereby improve the health of the population [1], it is important to identify the role of medical education in preparing Indonesia’s doctors for the many public health challenges they will encounter across the diverse social and geographical environment of their workplace. Several medical institutions around the world have made successful efforts to improve the quality and the quantity of public health content in their medical curricula [11]. However, before this can be done in Indonesia, the current medical curricula need to be evaluated [11]. There are limited published reports of reviews of Indonesian medical curricula, and even fewer that have reviewed the public health curricula.

One full-time semester in Indonesian higher education equals a maximum of 24 credit points (160 minutes of learning per week) over 16 weeks [12]. In order to graduate with the title of “Dokter”, medical students must undertake at least seven semesters of course work (144 credit points) in the bachelor phase, and around two years of clinical placement in the clinical phase.

All Indonesian medical faculties base their medical school curriculum on the 2012 Indonesian Standard for Doctor Competency (Standar Kompetensi Dokter Indonesia (SKDI 2012)) [13]. The SKDI includes public health amongst its competencies and lists 38 public health topics that can be included in the medical curriculum [13]. However, how many and which of the 38 topics each school includes as part of their medical degree is at their discretion, resulting in the possibility of great variation and inconsistency in curricula between the 86 medical schools across the country [14].

National medical school accreditation is carried out every five years and medical schools are categorized into three grades (A, B, or C), based on their performance in seven areas: (1) vision, mission, and strategies; (2) governance and quality assurance system; (3) student affairs and graduates’ employability; (4) faculty and administrative staff; (5) curricula and academic atmosphere; (6) finance and infrastructure; and (7) research, community outreach, and collaboration [15]. Within these seven areas, it is clear that accreditation does not specifically evaluate public health teaching or its importance and inclusion as a curriculum component [15]. Whilst some may consider the national competency exam to be a tool for assessing the quality of teaching [16], the public health content in the exam is relatively minor, and the multiple-choice question style of the examination may not adequately assess public health competency [16].

It is critical to ensure healthcare systems can adequately manage public health issues, and for medical professionals to have the capacity to be effective leaders and advocates for their communities, wherever they may be located. Indonesia is a large country both geographically and in a social sense. Well-trained medical graduates play a key role in the provision of healthcare and the implementation of disease prevention and health protection strategies. This paper explores how public health is represented in Indonesian medical curricula, and across the Eastern and Western geographical regions and accreditation grades.

## 2. Materials and Methods

A list of all medical schools across Indonesia was obtained from the Indonesian Medical Council [14] on 1 December 2019, and websites associated with these medical programs were identified through a search using Google^TM^. First, the full title of each medical school name was searched, for example, “Fakultas Kedokteran Universitas xxx”, (where xxx equals the University name), or well-known initials of the university, for example, “FK Universitas xxx” OR “FK Unxxx” OR “FKUxxx”. If no link to the medical school was found, the university name was searched, and the medical school link was found this way.

### 2.1. Inclusion Criteria

Medical schools were included if they were located in Indonesia, information on their medical curriculum was publicly available on their websites, and information was sufficient in terms of showing the name of all subjects in the bachelor stage and their size in credit points.

### 2.2. Data Extraction

The total credit points required for the undergraduate medical degree for each school was obtained from the relevant website, and then subject information was extracted. Curriculum information was found in a variety of forms, including a course syllabus, electronic curriculum books, academic calendars, dropdown menus, links to teaching materials (such as lecture slides, teaching modules, and/or tutorial sheets), medical school profiles, or downloadable Excel sheets.

The following data were extracted for each subject: subject name, number of credit points, bachelor or clinical phase, compulsory or elective subject, semester taught, duration (in weeks), subject description, and the date that the site was last updated. To ensure no subjects were missed, the subject credit points were then totaled and checked against the degree total credit points.

### 2.3. Analysis

The names and descriptions of the subject content were reviewed. Subjects with unclear information (for example, their names were provided only as unfamiliar initials, or information was in an unreadable picture form), and subjects that did not provide information on their size were excluded from the analysis. Subjects with the same name or the same content were regarded as the same subject. Subjects were not considered the same subject if different aspects of topics were taught in different subjects (for example, the physiological condition was covered in one subject and the pathological conditions in another).

The subjects were then categorized into groups. Researcher triangulation was used to increase the rigor and reduce the possibility of bias. First, the lead author, an Indonesian medical doctor and academic in medical education, reviewed the subject names and descriptions and broadly categorized subjects as either ‘biomedical/clinical’ subjects or ‘non-biomedical/nonclinical’ subjects. The lead author then categorized each subject into one of the five categories used by the 2012 Indonesian SKDI: (1) Biomedicine, (2) Clinical Science and Skills; (3) Social and Humanity Medicine, (4) Professionalism, and (5) Public Health. Subjects where content included both professionalism and any aspect of social issues or content that pertained to the humanities such as the arts, were categorized as (3) Social and Humanity Medicine [13]. A second, independent Indonesian medical doctor who is also an academic in medical education reviewed the classification and discrepancies were discussed and resolved by consensus where possible. Where consensus could not be reach, a third Indonesian academic who is the head of a medical education unit (MEU) was consulted to resolve any differences in categorization.

The null hypothesis was that there would be no difference in teaching of public health between medical schools in the Eastern and Western regions, or between accreditation levels of the medical schools. Descriptive statistical analyses were undertaken using the statistical software package, SPSS Version 25. The Shapiro–Wilk and Kolmogorov–Smirnov tests were used to test whether the data were normally distributed across geographical regions. Across accreditation levels, normality was also demonstrated. The Levene’s test was used to check the homogeneity of variance. Once parametric assumptions were checked and met, the results were further scrutinized using a two-way ANOVA with the significance level was set at *p* < 0.05. The two-way ANOVA test was chosen as it has higher power than non-parametric tests.

The percentages for each subject type within each course were compared across the dataset, and an analysis of the estimated marginal means was undertaken. This method allowed for the uneven sizes of the subject types and school data, and 95% confidence intervals were reported. Post hoc follow-up was applied to identify any differences.

Although variation in terms of the number of public health subjects and the extent of public health content was found in the bachelor stage, this variation was not obvious in the clinical stage (data not shown). Thus, this paper focuses only on the public health content identified in the bachelor stage of the degree.

## 3. Results

**Figure 1:**
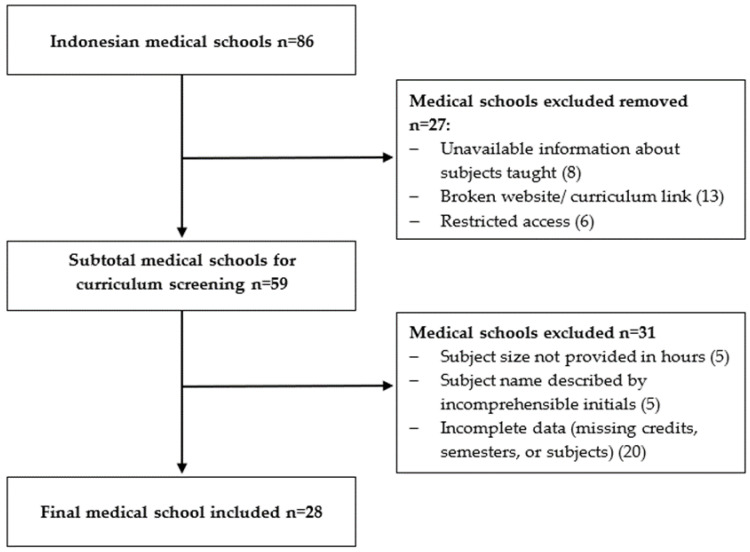
Flow chart of website search for information on medical degree bachelor stage subjects in the 86 Indonesian medical schools. Twenty-eight medical schools fit the inclusion criteria and were included in the analysis.

Twenty-eight medical schools fit the inclusion criteria and were included in the analysis (Figure 1).

**Figure 2:**
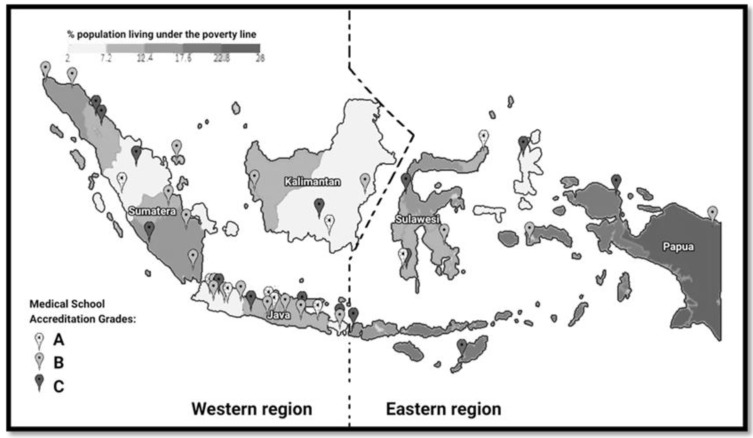
Distribution of all medical schools across the Eastern and Western regions of Indonesia and percent population living under the poverty line [14,17].

The 28 included medical schools were distributed across the Eastern and Western regions of Indonesia and included all accreditation grades (A, B, and C) (Figure 2). It should be noted that the dashed line dividing the Eastern and Western regions is not an official division and is solely for the purpose of demonstrating the spread of medical schools included in this study across the Eastern and Western regions [14,17].

Figure 2 also shows the poverty rating for the different geographical regionals of Indonesia, and highlights the well-recognized socio-economic gap between the Eastern and Western regions in Indonesia [18]. The poverty rating is based on the Indonesian poverty line, where a person is deemed to be living in poverty if they earn less than IDR 440,538 (USD 30.2) per month, or approximately USD 1 per day [17]. This amount of money is about half the global poverty rating determined by the World Bank (USD 1.90/day) [19].

Figure 2 demonstrates that there are more medical schools in the more urbanized Western region (n = 66) than in the Eastern region (n = 20). Moreover, there are more medical schools in the Western region with the higher accreditation grades of A (n = 19) and B (n = 33) than in the Eastern region. The curriculum analysis of the 28 Indonesian medical schools included in this study showed that all medical schools appear to be compliant in regards to the need for a minimum of 144 credits for the bachelor stage and the requirement to cover all five areas specified in the 2012 Indonesian Standard for Doctor Competency [12,13]. The size of each of the five subject groups of Indonesian medical curricula was very similar.

The number of subjects required in the bachelor phase in the 28 medical schools included in this analysis ranged from 30 to 57. After taking into account the various different subject names and combinations of content, 388 different individual subjects were identified. Table 1 shows the distribution of subjects across the five groups (Basic Biomedicine, Clinical Science and Skills, Social and Humanity Medicine, Professionalism, and Public Health) based on the Indonesian Standard of Medical Competency [13]. To ensure anonymity of schools, the ranges are provided for number of subjects and credits size.

**Figure 3:**
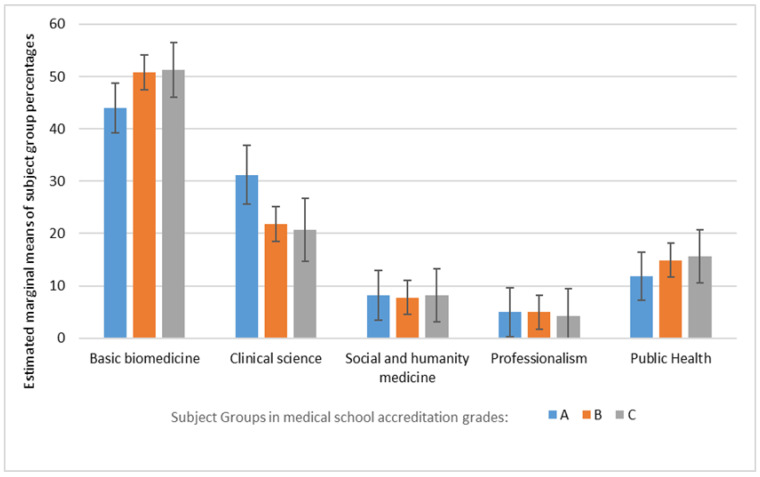
Estimated marginal means of subject grouping percentages in the bachelor phase of medical schools in Indonesia.

Table 1 shows that an Indonesian medical student is required to learn an average of 151.1 credits (SD 6.9) during the bachelor phase. Subjects in the Basic Biomedicine group represented the most frequently taught type of educational content (49.2 ± 8.7%), followed by Clinical Science (23.8 ± 8.6%) and Public Health (14.3 ± 5.0%) groups. Subjects in the Social and Humanity Medicine group (7.8 ± 4.4%) and in the Professionalism group (4.9 ± 2.0%) were represented the least. The curriculum profiles of schools with accreditation grades B and C were essentially the same across all the groups; however, schools in accreditation grade A appeared to have a greater emphasis on Clinical Science at the expense of Basic Biomedicine and Public Health, when compared to schools with accreditation grades of B and C. However, the results of the two-way ANOVA statistical analysis showed that all *p* values returned were higher than 0.05, which means there was no statistically significant difference between the percentages of subject types across the three medical program accreditation types (Figure 3).

**Figure 4:**
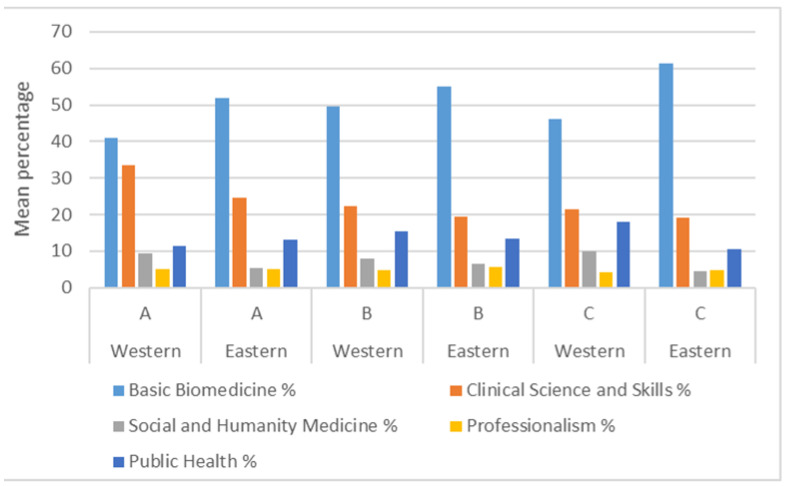
Comparison of curriculum topics according to accreditation grade (A, B, or C) and geographical location (Eastern or Western region).

When comparing the Western against the Eastern region medical schools (Figure 4), the curriculum profiles appear similar for the Western and Eastern regions for accreditation grades A and B, suggesting no influence of geographical region in the curricula taught. Although only six schools were included in the accreditation type C, Eastern region schools had statistically significantly (*p* = 0.035) more Basic Biomedicine subjects compared to the corresponding Western region schools with the same accreditation.

## 4. Discussion

Public health has been recognized as an important component of medical curricula across the world, and medical educators have expressed concern about the underrepresentation of public health in the curricula [1]. It is well recognized that medical schools find it hard to embed public health into the curriculum [11]. Whilst medical educators report that they find it relatively easier to teach biomedicine/clinical subjects as they can incorporate these into medical decision making, including improving patient well-being, doing this for public health topics such as the social determinants of health is not as easy [20]. Our findings show that Indonesia has done well in terms of the proportion of public health subjects taught in the curricula, with up to one-third of the curriculum content being filled by public health and related topics, regardless of the accreditation grade or geographical location of the school. This finding demonstrates not only that public health is considered an important feature in the Indonesian medical curricula, but also that the directions to include such material are being accommodated by medical school academics.

The significant representation of public health within Indonesian medical curricula is also likely to reflect Indonesia’s commitment to addressing its significant health priority issues [7,13]. The Eastern region is less developed and has higher rates of maternal deaths and childhood stunting, and lower doctor–patient ratios. One might then expect a higher representation of public health in the medical curricula in the region [18]. However, Figure 4 shows that only medical schools with accreditation grade A had more public health than their Western region counterparts. Furthermore, the schools with the highest percentage of biomedicine subjects were in the Eastern region with accreditation grade C. Schools of accreditation grade C are typically younger and in the process of recruiting the required academic and clinical staff to meet various accreditation requirements, and therefore recruiting medical educators who will teach clinical and biomedical subjects is prioritized [21]. Although the findings suggest public health as important feature in Indonesian medical curricula, several important events indicate that the public health curricula may need improving. Indonesian medical curricula have evolved based on a standard developed in 2012 [13]. Some studies have questioned the competency of Indonesian doctors in areas such as effective communication, creating inter-sectoral relationships, and effective leadership [22,23], particularly in Indonesian primary healthcare settings [24]. The Indonesian government determined that the current undergraduate medical curricula did not provide Indonesian doctors with sufficient public health competency [25] and introduced an additional specialty of family physician/ medicine specialists. This, like other specialties, is attained with additional three years of specialty training after the undergraduate program [26,27]. However, the Indonesian Medical Association has expressed their concern about this program stating, arguing that it would exacerbate the lack of doctors in the poorer Eastern regions of Indonesia [28] and that a focus on improving public health teaching in the current medical curriculum would be an acceptable alternative approach [29].

Frequent course content review is critical to ensure that important skills are taught effectively to medical students in Indonesia and elsewhere [22]. Further studies may review aspects such as how to improve teaching materials to make them more applicable, for example by designing materials that enable medical students to learn through more hands-on experiences when working within a community setting [22]. A system of peer support and review could also play a role for newer medical programs as they undergo changes to meet various accreditation requirements. In our study, there were some interesting results relating to schools belonging to the C accreditation grade, with a possible trend towards a different curriculum profile across the two geographical regions of Indonesia. Schools of accreditation grade C are typically younger and in the process of recruiting the required academic and clinical staffing [21]. In this context, frequent review would be helpful to ensure accreditation requirements to move these schools to a higher status are being addressed.

Analyzing the representation of public health within an entire medical curriculum is not easily done. Crucial information related to teaching is often hard to find as some academics regard the transparency on this area as a violation to their academic freedom [30]. Whilst only 28 schools were included in our study due to the study’s inclusion criteria, only 8 of the total 86 Indonesian medical schools did not make the content available through their websites, and a further 6 had password-protected information. The widely accessible information regarding Indonesian medical curricula suggests a positive, enabling environment for curriculum review, which other countries may want to adopt. Considering the included schools were well distributed across both the Eastern and Western regions of Indonesia and all accreditation grades, it is likely that the results are representative of all medical regions in Indonesia. This paper is important in a national context because it demonstrates both the transparency of Indonesian medical schools as well as their efforts to reflect global and local issues in their teaching. Comparing the representation of public health in the Indonesian medical curricula to that of other countries was outside the scope of this research but warrants further research in the future. In the Indonesian context, it would also be useful to explore the detail of the materials provided within each public health subject, and how much, and to match these with the 38 topics in the 2012 Indonesian national competency standard. Moreover, the findings reported here can be the first step for national medical curriculum mapping in Indonesia, and be a catalyst for supporting medical educators to exchange information about what is being taught and coordinate it to reflect the health priorities issues particularly in the Indonesian public health and community health context [22]. Exploiting advancements in information and technology, it is now possible for LMIC to develop a web-based interactive platform for medical curriculum mapping that would allow medical schools to visualize how their curricula compares to given national competency standards [31]. There is potential for further improvements in curriculum design by looking more deeply into the need to incorporate curricular elements including people (students and educators), topics, activities (learning and assessment events), learning outcomes and objectives, and learning resources [32]. In this context, it will also be important for educators to embrace the current trend towards competency-based assessment, and to ensure that graduates’ understanding and competence in public health topics relevant to the Indonesian setting are demonstrated.

This study has some limitations. Due to the level of information available on the schools’ websites, it was not possible to check the actual content taught in each subject. Subjects could have been incorrectly categorized into the wrong subject group. However, the rigorous way in which each subject’s information was reviewed by a team of three academics with expertise in the Indonesian medical curricula helped minimize this possibility. The curriculum mapping was performed on publicly available information on medical school websites, and it is possible that as the information on some medical schools may not have been up to date, even though updates on medical school websites throughout the duration of data collection were carefully monitored and re-checked at the end of the extraction dates. Due to funding and time constraints, it was not possible to contact each school individually. The sample size to check significant differences between accreditation grades C in the Western schools was small (six schools), and results should be viewed with caution.

## 5. Conclusions

Public health competency is critical for medical doctors, and medical schools need to ensure that their public health curricula reflect global and local public health issues are flexible to the future challenges of the changing world and are applicable to the context in which doctors work. Curriculum review and mapping are central for this effort, and this can be done in multiple ways, including by reviewing the representation of subjects within the whole medical curricula, as was done in this study. Indonesian medical schools are doing well in regard to curriculum transparency and those included in this study had public health as an important feature in Indonesian medical curricula. The level of public health featured in the curricula is generally consistent across the included medical schools regardless of their geographic location (Eastern or Western Indonesia) and accreditation grade. Further research is critical to explore aspects such as the materials taught, the relevance and the applicability of the specific public health content, and the assessment of public health competency of graduates.

## Figures and Tables

**Table 1:** Distribution of accreditation grade, educational group, and subject size of included medical schools in Indonesia.

	Medical School Code	No. Subjects in Bachelor Phase	No. of Credits in Bachelor Phase	Basic Biomedicine	Clinical Science and Skills	Social and Humanity Medicine	Professionalism	Public Health
				%	%	%	%	%
**Medical school** **accreditation grade A**	WA1	30–39	144–154	44.4	29.4	7.2	6.5	12.4
	WA2	30–39	144–154	33.3	31.3	7.5	2.7	25.2
	WA3	30–39	155–165	50	30	3.1	6.9	10
	WA4	50–59	166–176	42.3	28.6	21.7	7.4	0
	WA5	30–39	144–154	34.5	48.7	6.8	1.4	8.8
	EA1	40–49	155–165	44.8	24.5	8.6	6.1	16
	EA2	30–39	144–154	58.9	24.7	2.1	4.1	10.3
	Mean	38.7	156	44	31	8.1	5	11.8
	SD	6.1	10.6	8.8	8.2	6.5	2.3	7.6
**Medical school** **accreditation grade B**	WB1	40–49	144–154	55.2	11.7	14.9	5.8	12.3
	WB2	30–39	144–154	56.9	15.3	11.1	6.9	9.7
	WB3	40–49	144–154	44.6	31.1	12.2	4.1	8.1
	WB4	30–39	144–154	53.7	20.1	2.7	6.7	16.8
	WB5	50–59	144–154	50.3	24.8	7.4	1.3	16.1
	WB6	50–59	144–154	52	15.3	11.3	8	13.3
	WB7	40–49	144–154	40.5	21.6	8.8	5.4	23.7
	WB8	30–39	144–154	56	14.7	5.3	6.7	17.3
	WB9	30–39	144–154	41.7	36.1	4.2	5.6	12.5
	WB10	40–49	144–154	56.5	18.2	6.5	2.6	16.2
	WB11	30–39	144–154	57.4	16.9	6.8	2.7	16.2
	WB12	40–49	144–154	30.5	41.7	2.7	2.7	22.5
	EB1	40–49	144–154	50	20.6	6.2	8.9	14.4
	EB2	30–39	155–165	60.7	17.4	4.5	2.6	14.8
	EB3	40–49	144–154	54.3	20.3	9.2	5.2	11.1
	Mean	43.1	149.5	50.7	21.7	7.6	5	15
	SD	7.5	3.4	8.1	8.4	3.6	2.3	4.2
**Medical school** **accreditation grade C**	WC1	30–39	144–154	52.1	25	5.6	4.2	13.2
	WC2	40–49	144–154	50	21	11.5	3.4	14.2
	WC3	40–49	155–165	44.4	24.7	9.3	4.3	17.3
	WC4	40–49	144–154	38.5	15.5	13.5	4.7	27.7
	EC1	30–39	144–154	63.8	13.4	6.7	5.4	10.7
	EC2	30–39	144–154	58.9	24.7	2.1	4.1	10.3
	Mean	40.7	149.5	51.3	20.7	8.1	4.3	15.6
	SD	6.7	6.4	9.2	5.1	4.2	0.7	6.5

Key: W—Western region of Indonesia; E—Eastern region of Indonesia; A, B, and C—medical school accreditation grade.

## Data Availability

The raw data are not publicly available to protect the identity of individual medical schools. Aggregated data for this study are available from the lead author on reasonable request.
